# Comparative Effectiveness of Coronary CT Angiography and Standard of Care for Evaluating Acute Chest Pain: A Living Systematic Review and Meta-Analysis

**DOI:** 10.1148/ryct.230022

**Published:** 2023-08-24

**Authors:** Maurício F. Barbosa, Arzu Canan, Yin Xi, Harold Litt, Deborah B. Diercks, Suhny Abbara, Fernando U. Kay

**Affiliations:** From the Department of Radiology, Cardiothoracic Division (M.F.B., A.C., S.A., F.U.K.), Department of Radiology (Y.X.), and Department of Emergency Medicine (D.B.D.), UT Southwestern Medical Center at Dallas, 5323 Harry Hines Blvd, Dallas, TX 75390; and Department of Radiology, University of Pennsylvania, Philadelphia, Pa (H.L.).

**Keywords:** Acute Coronary Syndrome, Chest Pain, Emergency Department, Coronary Computed Tomography, Usual Care

## Abstract

**Purpose:**

To perform a living systematic review and meta-analysis of randomized controlled trials comparing the effectiveness of coronary CT angiography (CCTA) and standard of care (SOC) in the evaluation of acute chest pain (ACP).

**Materials and Methods:**

Multiple electronic databases were systematically searched, with the most recent search conducted on October 31, 2022. Studies were stratified into two groups according to the pretest probability for acute coronary syndrome (group 1 with predominantly low-to-intermediate risk vs group 2 with high risk). A meta-regression analysis was also conducted using participant risk, type of SOC used, and the use or nonuse of high-sensitivity troponins as independent variables.

**Results:**

The final analysis included 22 randomized controlled trials (9379 total participants; 4956 assigned to CCTA arms and 4423 to SOC arms). There was a 14% reduction in the length of stay and a 17% reduction in immediate costs for the CCTA arm compared with the SOC arm. In group 1, the length of stay was 17% shorter and costs were 21% lower using CCTA. There was no evidence of differences in referrals to invasive coronary angiography, myocardial infarction, mortality, rate of hospitalization, further stress testing, or readmissions between CCTA and SOC arms. There were more revascularizations (relative risk, 1.45) and medication changes (relative risk, 1.33) in participants with low-to-intermediate acute coronary syndrome risk and increased radiation exposure in high-risk participants (mean difference, 7.24 mSv) in the CCTA arm compared with the SOC arm. The meta-regression analysis found significant differences between CCTA and SOC arms for rate of hospitalization, further stress testing, and medication changes depending on the type of SOC (*P* < .05).

**Conclusion:**

The results support the use of CCTA as a safe, rapid, and less expensive in the short term strategy to exclude acute coronary syndrome in low- to intermediate-risk patients presenting with acute chest pain.

**Keywords:** Acute Coronary Syndrome, Chest Pain, Emergency Department, Coronary Computed Tomography, Usual Care

*Supplemental material is available for this article.*

Published under a CC BY 4.0 license.

**Summary**

The use of coronary CT angiography to evaluate individuals with low-to-intermediate risk for acute chest pain was associated with shorter length of emergency department and hospital stay and reduced immediate costs.

**Key Points**

■ Coronary CT angiography (CCTA) demonstrated effectiveness as a safety strategy for evaluation of participants presenting with acute chest pain, showing similar incidence of myocardial infarction (relative risk, 0.86; 95% CI: 0.66, 1.12), all-cause mortality (relative risk, 0.96; 95% CI: 0.59, 1.58), and cardiovascular mortality (relative risk, 1.35; 95% CI: 0.59, 3.09), compared with usual care, irrespective of pretest probability.
■ The number of referrals for invasive coronary angiography after CCTA was not statistically different from standard of care irrespective of pretest probability. However, there were more revascularizations (relative risk, 1.45; 95% CI: 1.09, 1.93) and changes in medication (relative risk, 1.33; 95% CI: 1.06, 1.67) in participants with low-to-intermediate risk of acute coronary syndrome and increased radiation exposure (mean difference, 7.24 mSv; 95% CI: 4.55, 9.94) in higher-risk participants in the CCTA arm.
■ The use of CCTA in low- to intermediate-risk participants was associated with a 17% reduction in length of stay and a 21% decrease in immediate costs.

## Introduction

Acute chest pain (ACP) is the second most common reason for adult patients to visit the emergency department (ED) in the United States, accounting for approximately 6.3% of all ED consultations (1). While a small portion of these patients will have acute coronary syndrome (ACS) as the underlying cause of their ACP, the serious consequences of missed diagnoses and nonspecific clinical manifestation pose a challenge to ED services for triaging such patients (2,3). Therefore, providers usually follow a cautious approach to ACP, frequently including a combination of close clinical observation, electrocardiography, serial cardiac biomarkers, and stress testing, which has contributed to the increasing use of health care resources (2). More recently, the American College of Cardiology and the American Heart Association jointly published the guideline for the evaluation and diagnosis of ACP to address heterogeneity of practice among health care institutions (4). This guideline incorporates best practices based on accumulated evidence, including the role of emerging diagnostic tests such as coronary CT angiography (CCTA).

CCTA is a noninvasive imaging method with high accuracy for diagnosing obstructive coronary artery disease (CAD). CCTA’s utility is driven by its high sensitivity and negative predictive value (5). Previous meta-analyses corroborate the safety of CCTA compared with the standard of care (SOC) in the evaluation of ACP (6–9) suggesting the potential for reductions in use of health care resources as measured by length of ED and hospital stays (LOS) and overall costs. However, recent randomized controlled trials (RCTs) failed to reproduce those results (10–12). Reconciliation of these conflicting data is imperative to consolidate the strategic role of CCTA for assessing ACP (4).

Living systematic reviews (LSRs) are tools for incorporating novel evidence longitudinally, even after the initial publication of a manuscript and especially in fields where there is rapidly emerging evidence and when pending uncertainties exist (13). Our goal is to perform an LSR to evaluate the comparative effectiveness of CCTA versus SOC in the evaluation of ACP. We specifically focus on differences in resource utilization, clinical events, and survival. This LSR will continually update the data as new studies are published.

## Materials and Methods

### Literature Search and Study Selection

The Nested Knowledge living review platform (*www.nested-knowledge.com*) was used to perform this LSR and meta-analysis following the Preferred Reporting Items for Systematic Reviews and Meta-Analyses (PRISMA) guidelines (14). The electronic databases PubMed, Cochrane Library, Web of Science, Embase, Scopus, Google Scholar, and ScienceDirect were systematically searched for RCTs comparing CCTA and SOC. SOC procedures included but were not limited to history taking, physical examination, electrocardiography, biomarkers, and stress testing in the evaluation of adult participants with ACP. The last search for inclusion of new studies was conducted on October 31, 2022. The querying terms and respective search logic can be found in Appendix S1. Additionally, we searched the references of all included studies to identify potentially missed articles by the database searches.

After conducting the literature search, two independent readers (M.F.B. and A.C., cardiothoracic radiologists with 15 and 9 years of experience, respectively) screened the studies for inclusion, reviewing the title, abstract, and when necessary, the full text of the manuscript. Randomized trials published in peer-reviewed journals evaluating the effects of CCTA versus SOC on clinical outcomes and resource utilization in adult participants with ACP were included. Observational studies, abstracts, editorials, case series, and case reports were excluded. No language restriction was enforced. All disagreements were adjudicated by a third independent reader (F.U.K., cardiothoracic radiologist with 10 years of experience). To ensure the living component of our LSR, we plan to review the literature at least twice a year, so we will actively seek and incorporate new evidence as it becomes available.

### Data Extraction and Effect Measures

All data were collected from the published manuscripts and supplemental materials available online and inputted in the extraction module of Nested Knowledge. One author (M.F.B., cardiothoracic radiologist with 15 years of experience) abstracted data related to participant characteristics, including age, sex, race and ethnicity, body mass index, and cardiovascular risk factors (hypertension, hyperlipidemia, diabetes, smoking history, and family history of CAD), as well as outcomes, including LOS, number of invasive coronary angiographic (ICA) examinations performed, rate of revascularization, myocardial infarction (MI), all-cause mortality, cardiovascular mortality, time to diagnosis, further stress testing, repeat visits or hospitalizations, rate of hospitalization, heart failure, cardioembolic stroke, changes in medication, radiation exposure, participant satisfaction, and costs. Revascularization was defined as the sum of percutaneous coronary intervention and coronary artery bypass graft. Costs were converted to U.S. dollars using the market quotation on the extraction day. In instances of overlapping outcome data from the same population, we prioritized the longer follow-up period when analyzing hard clinical events such as MI and mortality. For all other data, we extracted information from the first published article. A second author (A.C., cardiothoracic radiologist with 9 years of experience) reviewed and validated all extracted data. Detailed results of this study search, screening, and data extraction process are hosted on the Nested Knowledge website (*https://nested-knowledge.com/nest/912*) (Fig S1).

### Study Risk of Bias and Certainty Assessment

Two authors (M.F.B. and F.U.K., cardiothoracic radiologists with 15 and 10 years of experience, respectively) scored the risk of bias for each study using the Cochrane Risk of Bias 2 (RoB 2) tool (15) and the certainty of the evidence using the Grading of Recommendations Assessment, Development, and Evaluation (GRADE) system (16). Disagreements were resolved by consensus.

### Data Synthesis and Publication Bias

Pooled relative risks and corresponding 95% CIs were calculated for binary clinical outcomes using random-effects models. Difference in means or ratio of means and 95% CIs were calculated for numerical continuous outcomes (LOS and costs were calculated with ratio of means and the radiation dose was calculated with difference in means), also using random-effects models. To understand the effects of different pretest probability on the pooled effects, we stratified the studies into two groups. Group 1 contains RCTs including study samples with predominantly low-to-intermediate risk for ACS, while group 2 is composed of RCTs including participants with a higher risk for ACS. We chose a 10% prevalence of high-risk participants in the study sample according to the definition of ACS risk chosen by each study as the classification criterion to differentiate between groups 1 and 2. Our decision to use this particular cutoff point was based on the discrepant outcomes observed in the Cardiac CT in the Treatment of Acute Chest pain (CATCH) trial (17) in comparison to the American College of Radiology Imaging Network-Pennsylvania (ACRIN-PA) (18) and Multicenter Study to Rule Out Myocardial Infarction by Cardiac Computed Tomography (ROMICAT-II) (19) trials. The prevalence of high-risk participants in the CATCH trial was approximately 10% which was higher than the other trials due to variations in eligibility criteria resulting in a greater prevalence of CAD among participants. Additionally, we designated any RCT that exclusively included participants with non-ST-segment elevation MI or elevated high-sensitive troponins as a high-risk cohort. Heterogeneity was assessed using Higgins and Thompson *I*^2^ statistic. *I*^2^ is the proportion of total variation observed between the trials attributable to differences between trials rather than sampling error (chance), with *I*^2^ values of less than 25%, between 25% and 75%, and greater than 75% corresponding to low, moderate, and high levels of heterogeneity, respectively. To assess the presence of publication bias, we employed a combination of visual inspection of funnel plots and conducted Egger tests for funnel plot asymmetry. This analysis was conducted for outcomes where a minimum of 10 studies were available. Finally, we conducted a meta-regression analysis, stratifying studies by patient risk category (group 1 vs group 2), type of SOC employed (ie, further testing at physician’s discretion vs routine stress echocardiography or nuclear medicine stress perfusion), and the routine use versus no use of high-sensitivity troponins as independent variables. All analyses were done with R software (version 4.2.1; The R Foundation) with package meta version 5.5-0 (20). A *P* value less than .05 indicated a statistically significant difference.

## Results

### Study Selection and Characteristics

**Figure 1:**
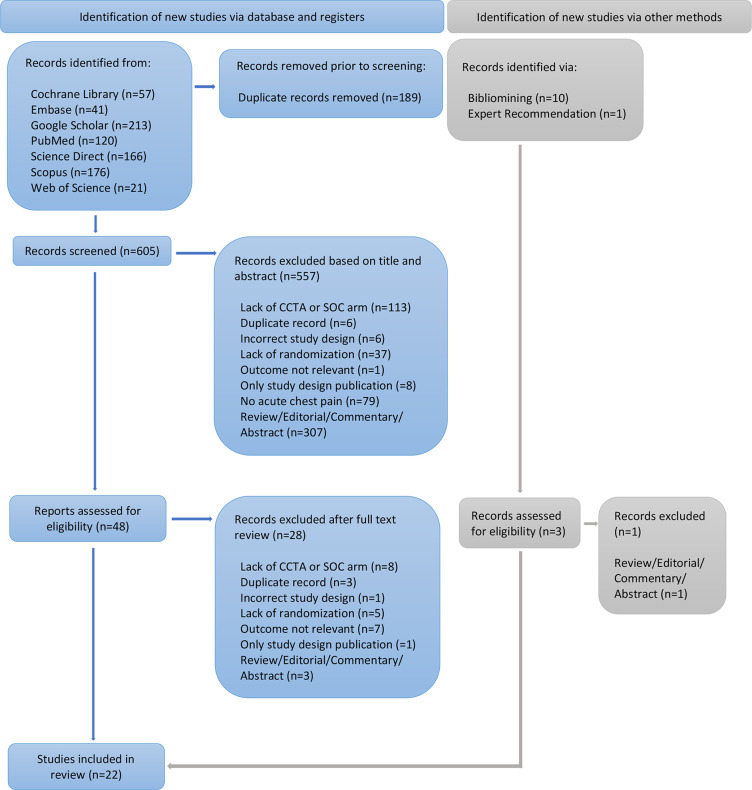
Preferred Reporting Items for Systematic Review and Meta-Analysis (PRISMA) flowchart demonstrates the screening process for identification of studies included. CCTA = coronary CT angiography, SOC = standard of care.

The results of the literature search are presented in Figure 1. After the exclusion of duplicated study entries, a total of 616 studies remained for screening. During the screening process, 565 studies were excluded based on title and abstract review, resulting in 51 articles for full-text review. Then, 29 studies were excluded because of lack of intervention or control arm; duplicated reports of the same research; incorrect study design (eg, not randomized); lack of relevant outcome; publication reporting only the study design; or because it was a review, editorial, commentary, or abstract. Finally, 22 RCTs (10–12,17–19,21–36) were included in the final analysis, representing a total of 9379 participants, with 4956 participants assigned to the CCTA arms and 4423 participants assigned to the SOC arms. The follow-up length ranged from 28 days to more than 5 years among studies. The main characteristics of the studies are summarized in Tables 1 and 2.

**Table 1:**
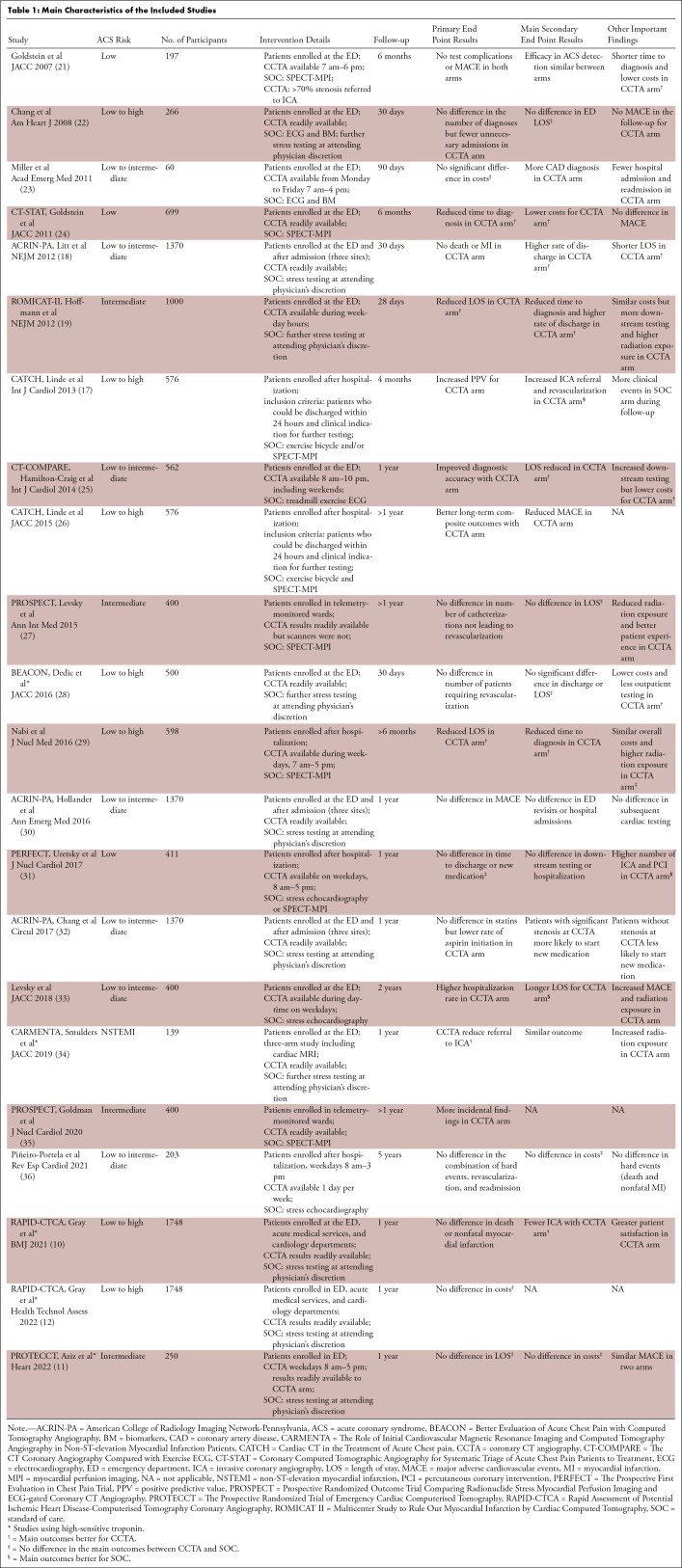
Main Characteristics of the Included Studies

**Table 2:**
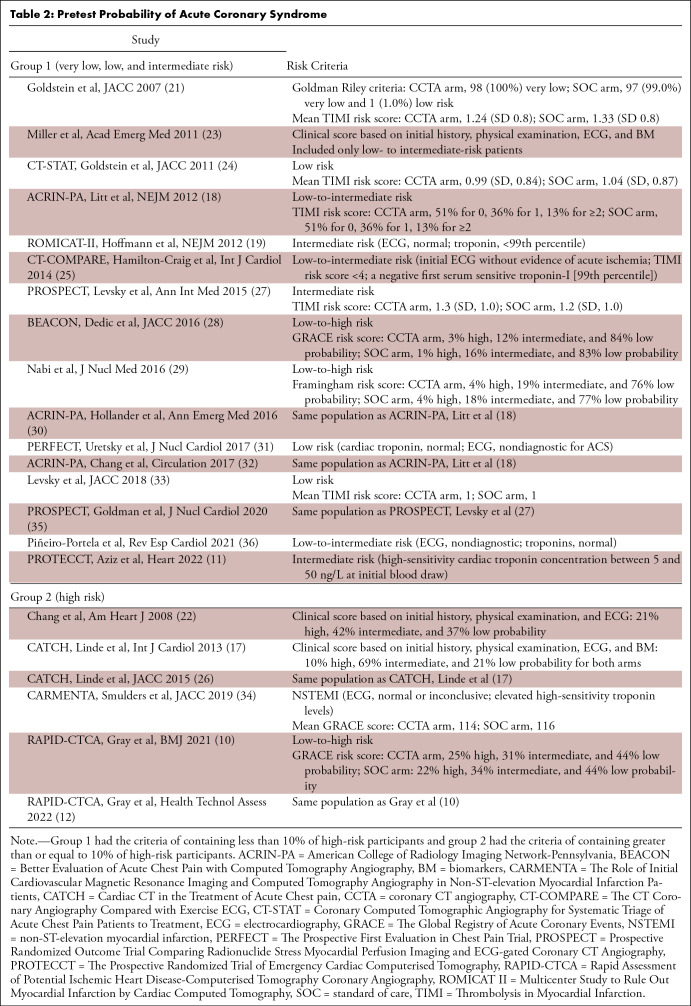
Pretest Probability of Acute Coronary Syndrome

We found no evidence of a difference in the baseline patient demographic characteristics between CCTA and SOC arms, as listed in Table 3, although the prevalence of hyperlipidemia was slightly higher in the SOC arm in two studies (31,34). The mean age of all participants included was 55 years, with 5066 (54%) male participants and 4313 (46%) female participants. Table 4 serves as a summary of the key findings for the main outcomes.

**Table 3:**
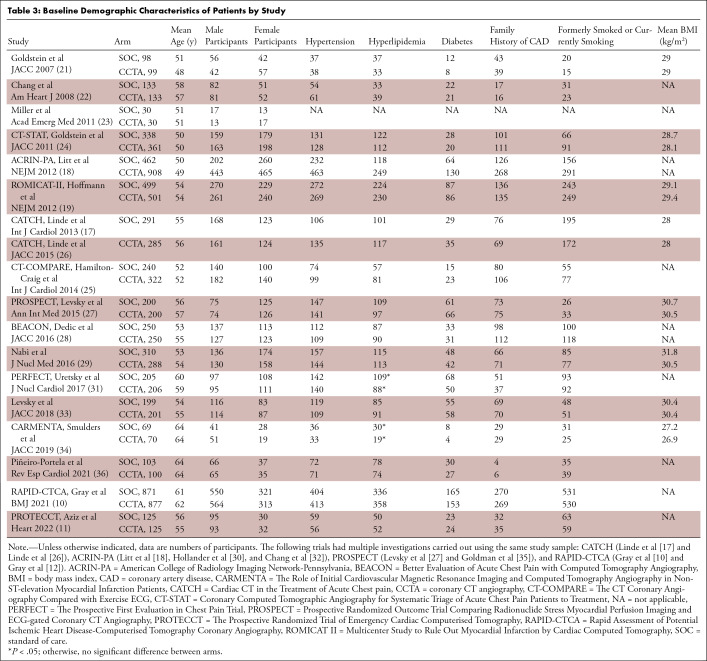
Baseline Demographic Characteristics of Patients by Study

**Table 4:**
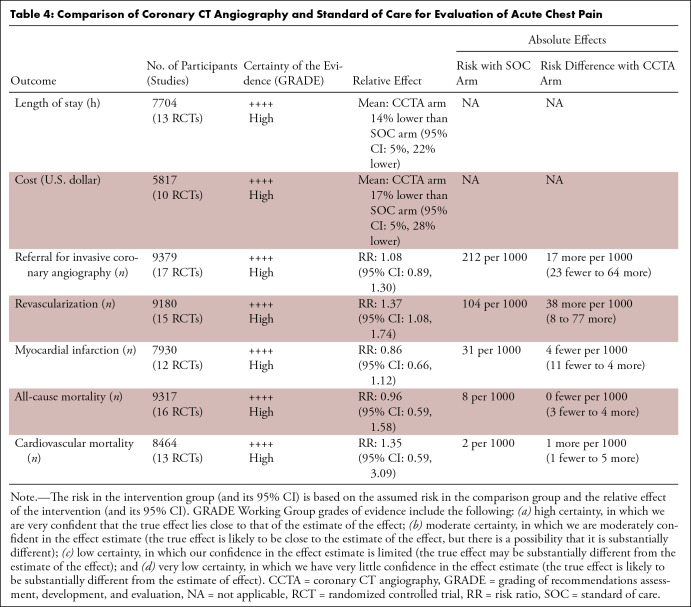
Comparison of Coronary CT Angiography and Standard of Care for Evaluation of Acute Chest Pain

### Length of Stay

**Figure 2:**
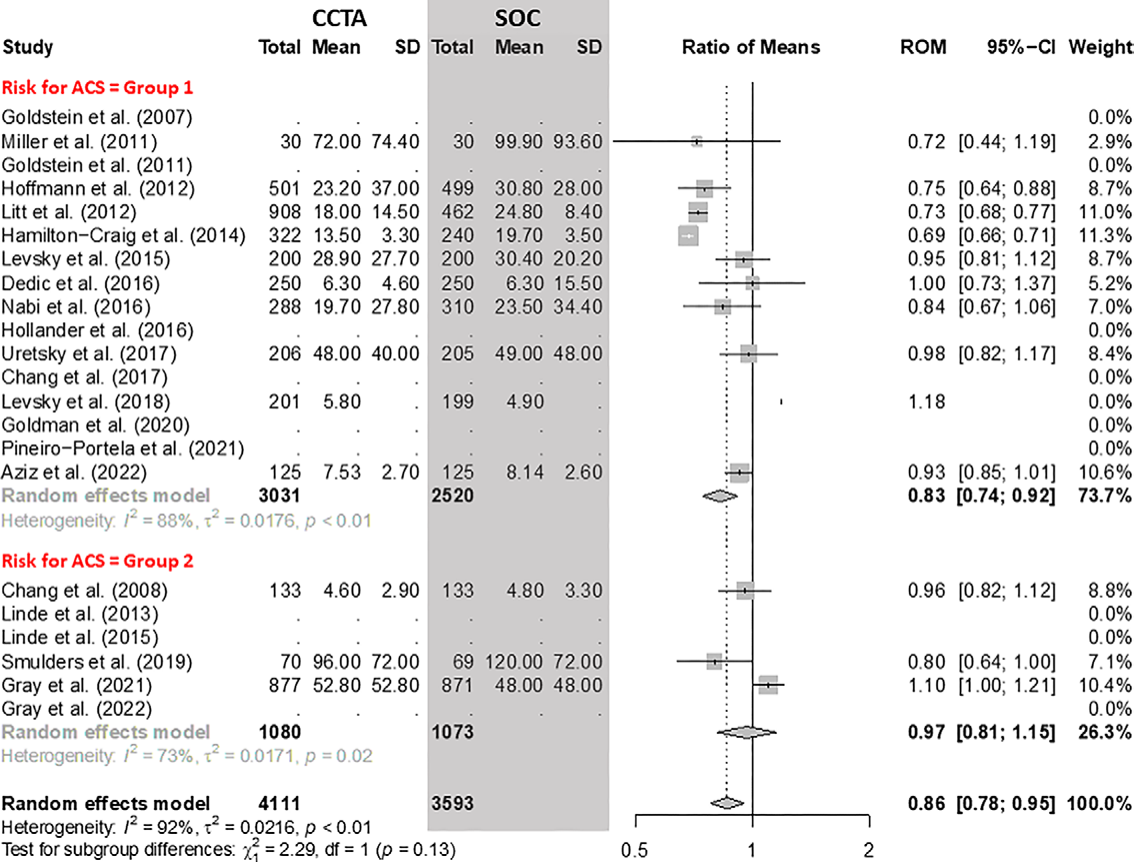
Comparison of the length of stay between coronary CT angiography (CCTA) and standard of care (SOC) arms. Forest plot shows the ratio of means (ROM) for length of stay (in hours) for CCTA compared with SOC arms in participants with acute chest pain, stratified by group (group 1 = low-to-intermediate risk for acute coronary syndrome [ACS] and group 2 = high risk for ACS). The overall ratio of means was 0.86 (95% CI: 0.78, 0.95). The size of central markers reflects the weight of each study. While all studies are listed, some of them have not studied all outcomes, which explains the missing values.

The pooled data showed a reduction of 14% (95% CI: 5%, 22%) in LOS for the CCTA arm compared with SOC arm (Fig 2). In group 1, considering the pooled data of 10 RCTs with 5551 participants, the LOS was 17% (95% CI: 8%, 26%) shorter following CCTA. However, in group 2, there was no evidence of a difference in the LOS between the two arms (ratio of means, 0.97; 95% CI: 0.81, 1.15).

### Referral for ICA

**Figure 3:**
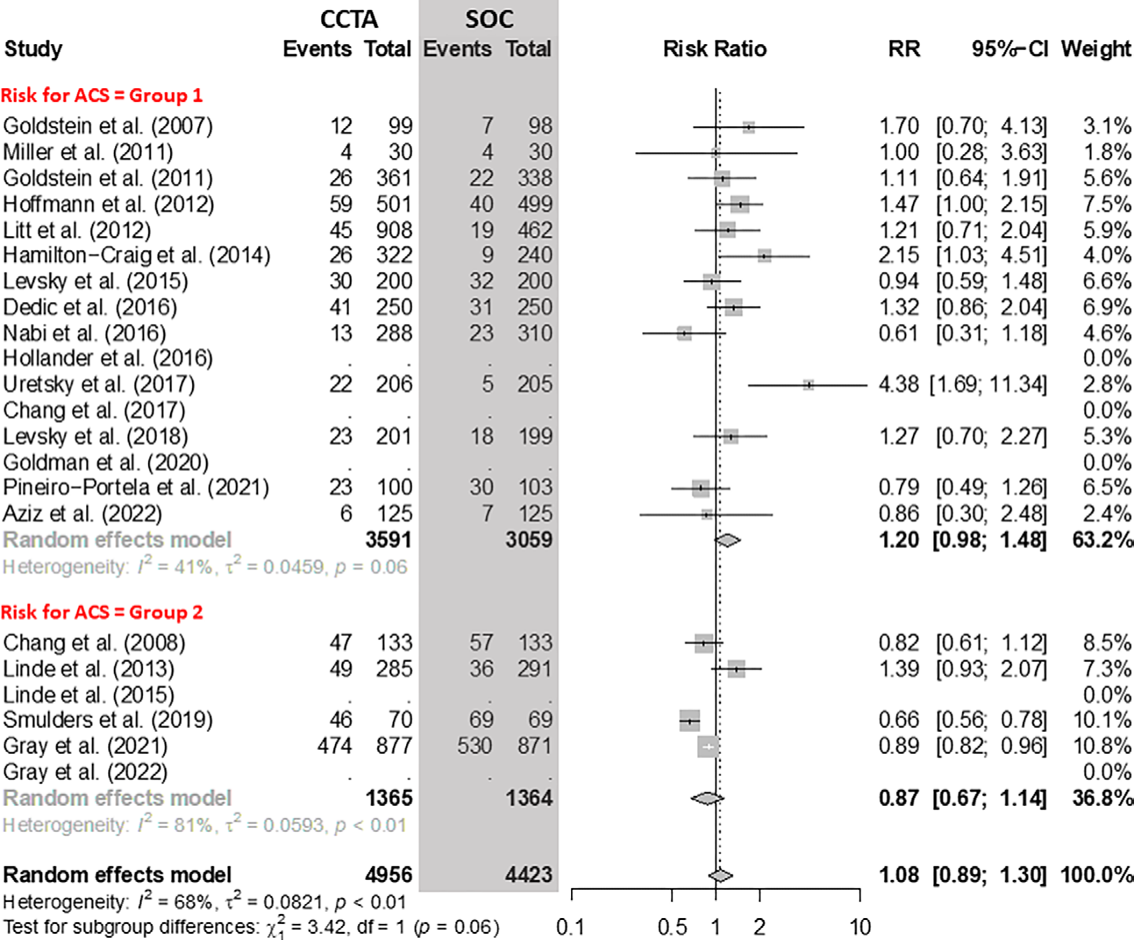
Comparison of the rate of invasive coronary angiography between coronary CT angiography (CCTA) and standard of care (SOC) arms. Forest plot shows the risk ratio (RR) of intensive coronary angiography for CCTA arms compared with SOC arms in participants with acute chest pain, stratified by group (group 1 = low-to-intermediate risk for acute coronary syndrome [ACS] and group 2 = high risk for ACS). The overall RR was 1.08 (95% CI: 0.89, 1.30). The size of central markers reflects the weight of each study. While all studies are listed, some of them have not studied all outcomes, which explains the missing values.

There was no evidence of a difference in the number of referrals for ICA between CCTA and SOC approaches (Fig 3). In group 1, considering 13 RCTs with 6650 participants, the risk ratio (RR) of ICA for CCTA versus SOC was 1.20 (95% CI: 0.98, 1.48). In group 2, considering four RCTs with 2729 participants, the RR of ICA for CCTA versus SOC was 0.87 (95% CI: 0.67, 1.14).

### Revascularization

**Figure 4:**
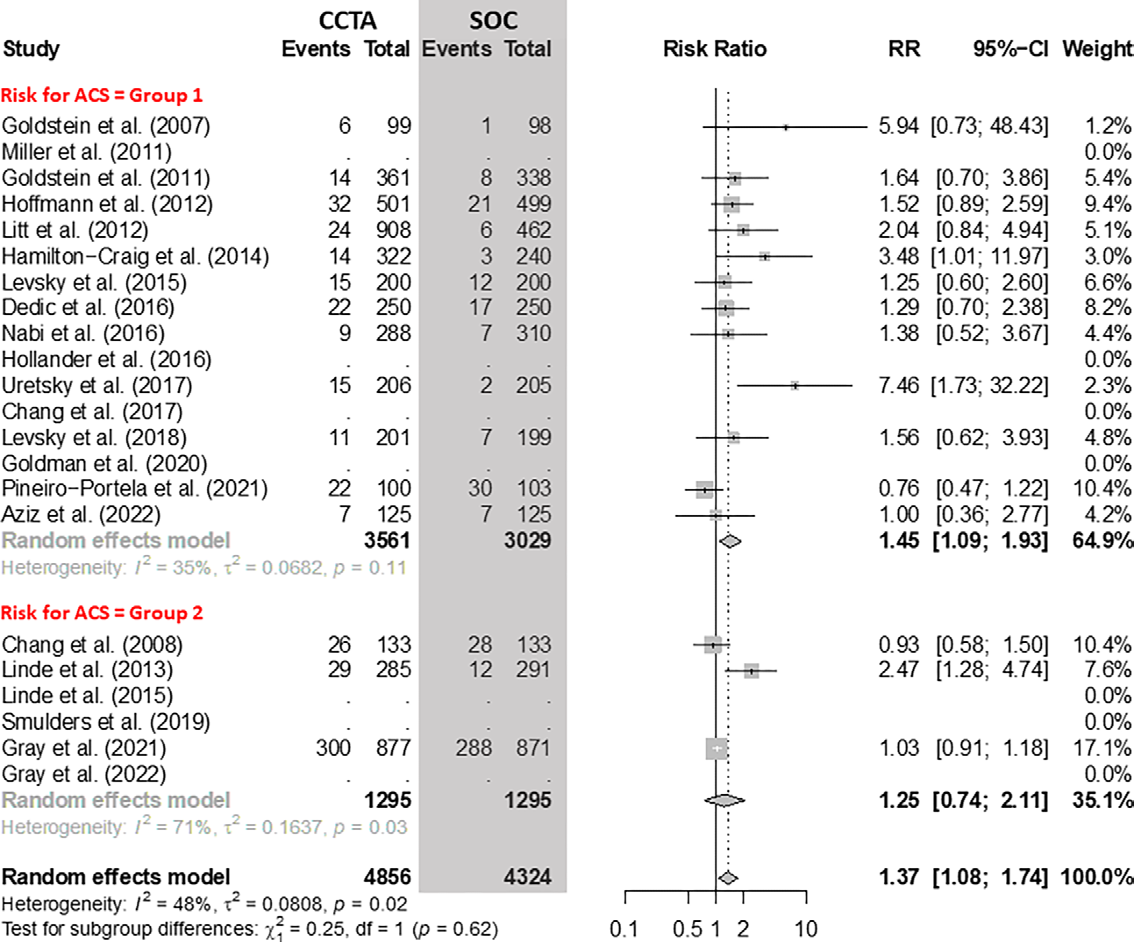
Comparison of the rate of revascularization between coronary CT angiography (CCTA) and standard of care (SOC) arms. Forest plot shows the risk ratio (RR) of revascularization for CCTA arms compared with SOC arms in participants with acute chest pain, stratified by group (group 1 = low-to-intermediate risk for acute coronary syndrome [ACS] and group 2 = high risk for ACS). The overall RR was 1.37 (95% CI: 1.08, 1.74). The size of central markers reflects the weight of each study. While all studies are listed, some of them have not studied all outcomes, which explains the missing values.

There were more revascularizations after CCTA compared with the SOC (Fig 4). The overall absolute increase of revascularizations after CCTA was 38 per 1000 participants (95% CI: 8, 77). In group 1, including 12 RCTs with 6590 participants, the RR of revascularization for CCTA versus the SOC was 1.45 (95% CI: 1.09, 1.93). In group 2, including three RCTs with 2590 participants, the RR of revascularization for CCTA versus the SOC was 1.25 (95% CI: 0.74, 2.11).

### Myocardial Infarction

**Figure 5:**
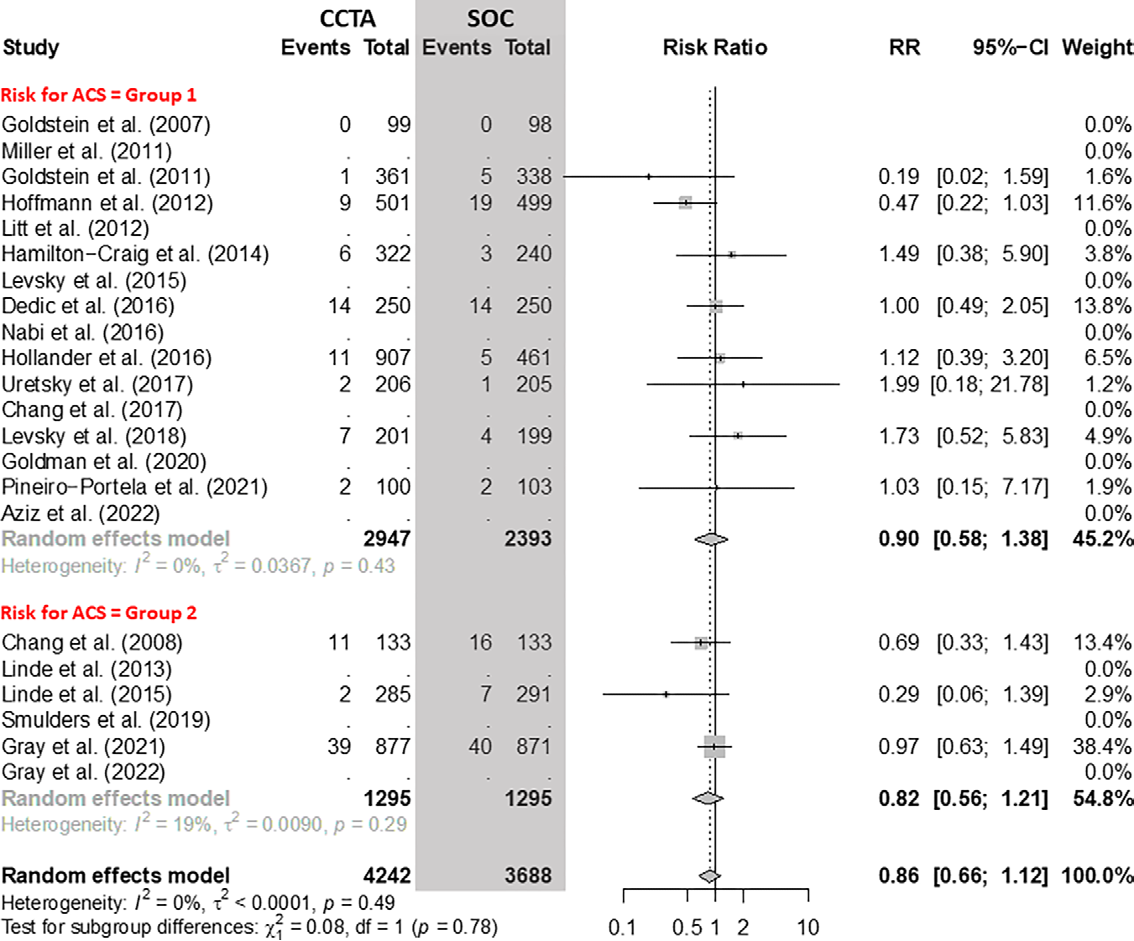
Comparison of the rate of myocardial infarction between coronary CT angiography (CCTA) and standard of care (SOC) arms. Forest plot shows the risk ratio (RR) of myocardial infarction for CCTA arms compared with SOC arms in participants with acute chest pain, stratified by group (group 1 = low-to-intermediate risk for acute coronary syndrome [ACS] and group 2 = high risk for ACS). The overall RR was 0.86 (95% CI: 0.66, 1.12). The size of central markers reflects the weight of each study. While all studies are listed, some of them have not studied all outcomes, which explains the missing values.

There was no evidence of a difference in the number of MIs between CCTA and SOC arms (Fig 5). In group 1, including nine RCTs with 5340 participants, the RR of MI for CCTA versus the SOC was 0.90 (95% CI: 0.58, 1.38). In group 2, including three RCTs with 2590 participants, the RR of MI for CCTA versus the SOC was 0.82 (95% CI: 0.56, 1.21).

### All-Cause Mortality

**Figure 6:**
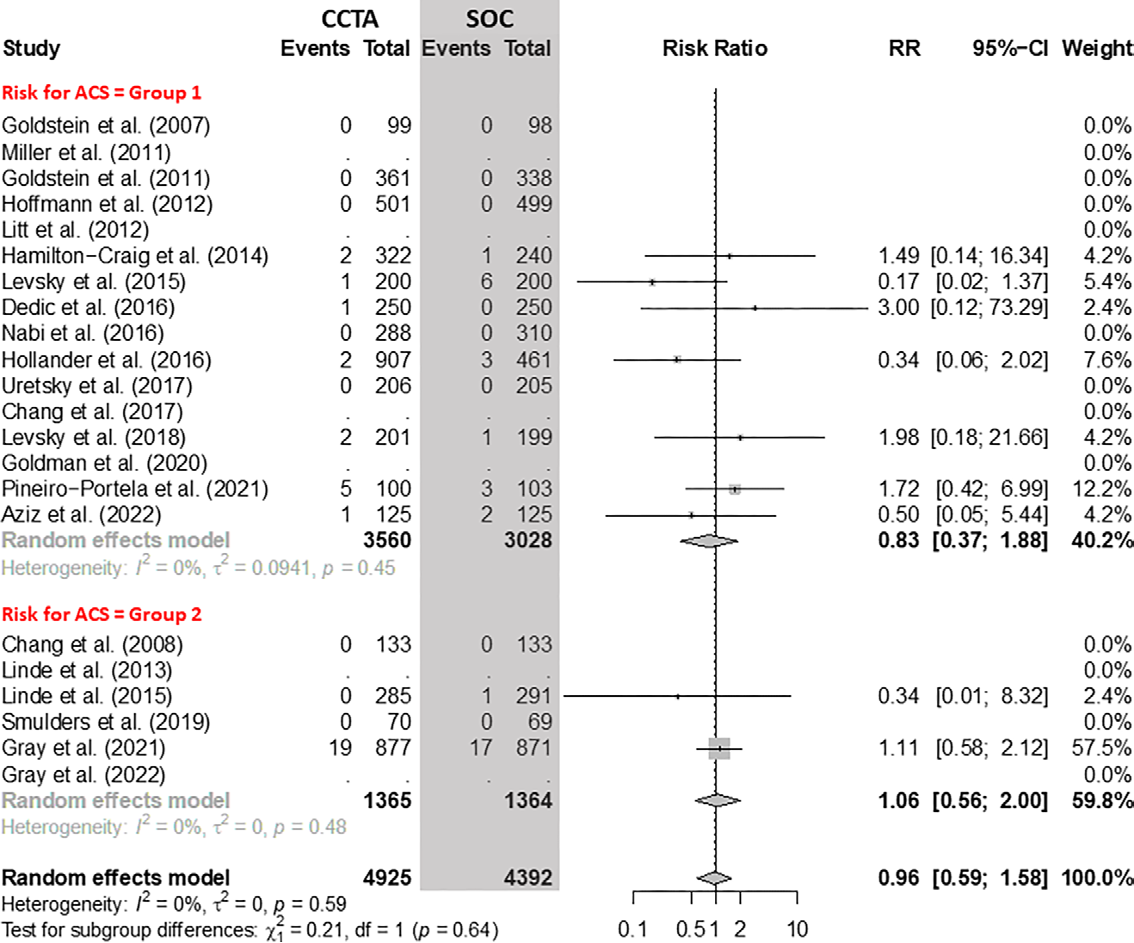
Comparison of all-cause mortality between coronary CT angiography (CCTA) and standard of care (SOC) arms. Forest plot shows the risk ratio (RR) of all-cause mortality for CCTA arms compared with SOC arms in participants with acute chest pain, stratified by group (group 1 = low-to-intermediate risk for acute coronary syndrome [ACS] and group 2 = high risk for ACS). The overall RR was 0.96 (95% CI: 0.59, 1.58). The size of central markers reflects the weight of each study. While all studies are listed, some of them have not studied all outcomes, which explains the missing values.

There was no evidence of a difference in all-cause mortality when comparing CCTA and SOC arms (Fig 6). In group 1, pooling 12 RCTs with 6588 participants, the RR of all-cause mortality for CCTA versus SOC was 0.83 (95% CI: 0.37, 1.88). In group 2, considering four RCTs with 2729 participants, the RR of all-cause mortality for CCTA versus SOC was 1.06 (95% CI: 0.56, 2.00).

### Cardiovascular Mortality

**Figure 7:**
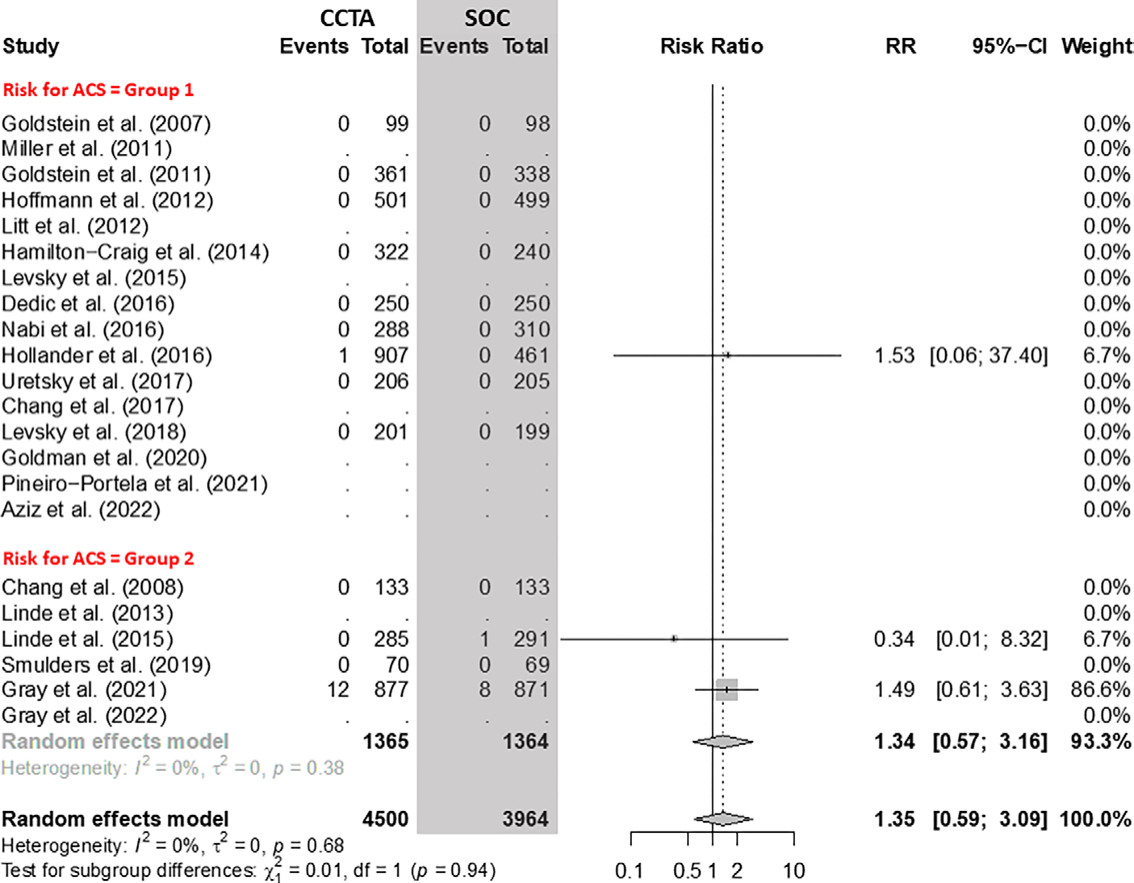
Comparison of cardiovascular mortality between coronary CT angiography (CCTA) and standard of care (SOC) arms. Forest plot shows the risk ratio (RR) of cardiovascular mortality for CCTA arms compared with SOC arms in participants with acute chest pain, stratified by group (group 1 = low-to-intermediate risk for acute coronary syndrome [ACS] and group 2 = high risk for ACS). The overall RR was 1.35 (95% CI: 0.59, 3.09). The size of central markers reflects the weight of each study. While all studies are listed, some of them have not studied all outcomes, which explains the missing values.

There was no evidence of a difference in cardiovascular mortality between CCTA and SOC arms (Fig 7). In group 1, nine RCTs with 5735 participants yielded a pooled RR for cardiovascular mortality of 1.53 (95% CI: 0.06, 37.40), while in group 2, four RCTs with 2729 participants yielded an RR of 1.34 (95% CI: 0.57, 3.16) between CCTA and SOC arms, respectively.

### Radiation Exposure

**Figure 8:**
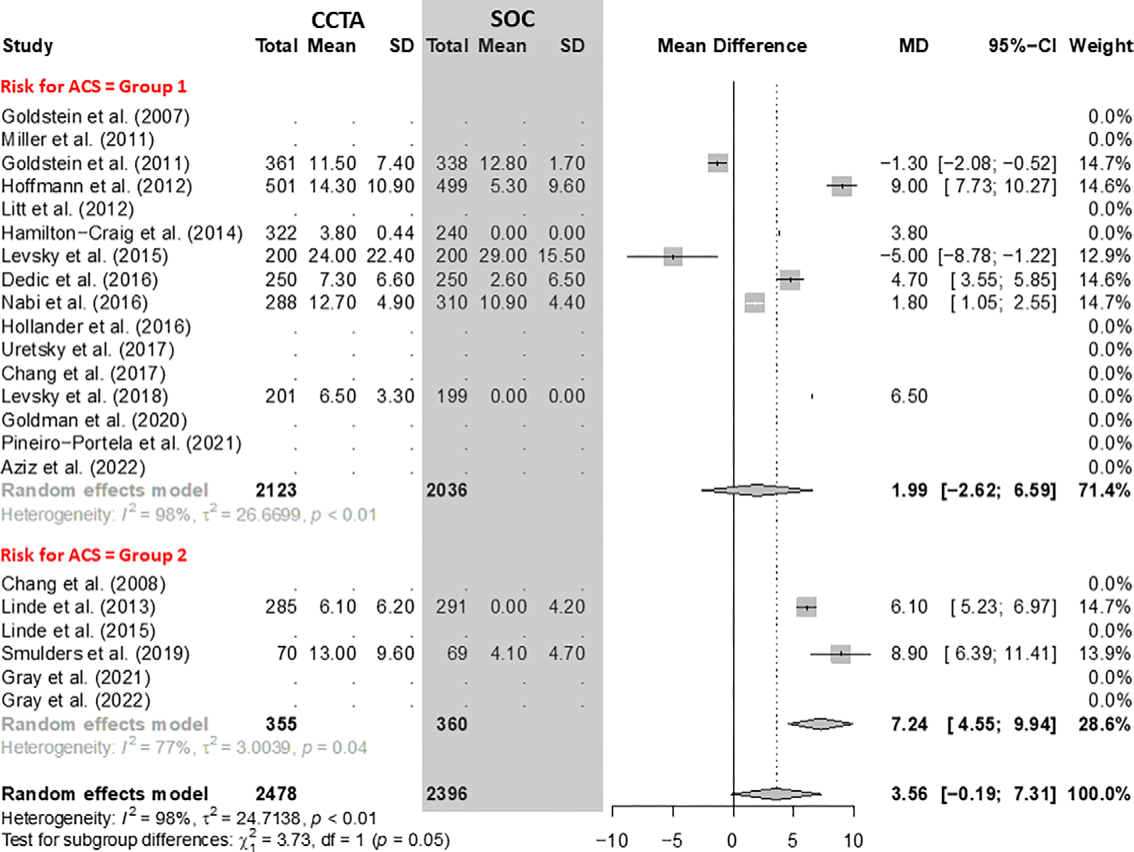
Comparison of radiation dose between coronary CT angiography (CCTA) and standard of care (SOC) arms. Forest plot shows the mean difference (MD) of radiation dose in millisieverts for CCTA arms compared with SOC arms in participants with acute chest pain, stratified by group (group 1 = low-to-intermediate risk for acute coronary syndrome [ACS] and group 2 = high risk for ACS). The overall MD was 3.56 (95% CI: -0.19, 7.31). The size of central markers reflects the weight of each study. While all studies are listed, some of them have not studied all outcomes, which explains the missing values.

Overall, there was no evidence of a difference in radiation exposure between CCTA and SOC arms. However, considering only group 2, the use of CCTA was associated with an increase in mean effective dose of 7.24 mSv (95% CI: 4.55, 9.94) when compared with SOC (Fig 8).

### Costs

**Figure 9:**
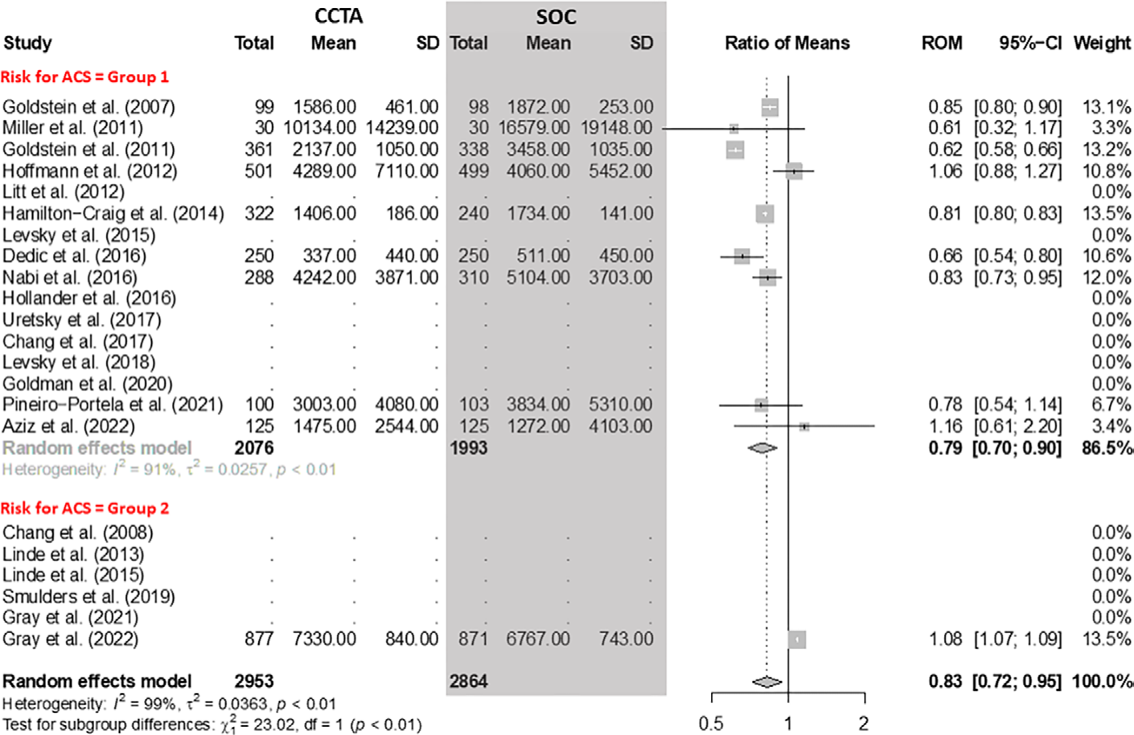
Comparison of costs between coronary CT angiography (CCTA) and standard of care (SOC) arms. Forest plot shows the ratio of means (ROM) for costs (U.S. dollars) for CCTA arms compared with SOC arms in participants with acute chest pain, stratified by group (group 1 = low-to-intermediate risk for acute coronary syndrome [ACS] and group 2 = high risk for ACS). The overall ROM was 0.83 (95% CI: 0.72, 0.95). The size of central markers reflects the weight of each study. While all studies are listed, some of them have not studied all outcomes, which explains the missing values.

The pooled data showed a reduction of 17% (95% CI: 5%, 28%) in costs when using CCTA compared with SOC (Fig 9). In group 1, considering the pooled data of nine RCTs with 4069 participants, the costs associated with CCTA were 21% lower (95% CI: 10%, 30%) in relation to SOC. For group 2, we identified only one RCT reporting costs in 1748 participants. In this study, the CCTA arm was associated with 8% higher (95% CI: 7%, 9%) costs compared with SOC.

### Rate of Hospitalization, Further Stress Testing, and Readmissions

There was no evidence of a difference in rate of hospitalization, further stress testing, and ED or hospital readmissions between CCTA and SOC approaches (Figs S2, S3, and S4, respectively).

### Changes in Medications

The analysis showed that overall, there were more instances of medication changes following CCTA compared with SOC (Fig S5). In group 1, consisting of five RCTs and a total of 2358 participants, the RR of medication change for CCTA versus SOC was 1.33 (95% CI: 1.06, 1.67). In group 2, with only one RCT including 1748 participants, the RR of medication change for CCTA versus SOC was 1.02 (95% CI: 0.95, 1.10).

### Incidental Findings

One study, a subanalysis of the Prospective Randomized Outcome Trial Comparing Radionuclide Stress Myocardial Perfusion Imaging and ECG-gated Coronary CT Angiography (PROSPECT) trial, reported more incidental findings in the CCTA arm compared with SOC arm (35). The authors reported 386 incidental findings in 187 participants who underwent CCTA. The most frequently occurring incidental findings at CCTA included pulmonary findings (118, 63%), noncoronary cardiac findings (69, 37%), gastrointestinal findings (49, 26%), hepatobiliary findings (42, 22%), and renal findings (17, 9%). No extracardiac incidental findings were noted at SPECT myocardial perfusion imaging studies. Also, there was a significantly higher frequency of incidental noncoronary inpatient medical workups in participants randomized to the CCTA arm compared with the SPECT myocardial perfusion imaging arm (20% vs 12%, *P* = .04).

### Meta-Regression

Our meta-regression analyses revealed three significant correlations, as shown in Table S1. When physicians had the discretion to determine the need for further stress testing, we observed a reduction in the rate of hospitalization and subsequent stress testing in the CCTA arm compared with the SOC arm (Figs S6 and S7, respectively). Also, we found that there were more medication changes in the CCTA arm compared with the SOC arm, particularly when SOC included stress echocardiography or nuclear medicine (Fig S8).

### Risk of Bias and Certainty of the Evidence

For the main desired outcomes, no study was judged as being at high risk of bias, as assessed by the RoB 2 tool, considering the following five domains: *(a)* randomization process, *(b)* deviations from the intended interventions, *(c)* missing outcome data, *(d) *measurement of the outcome, and *(e) *selection of the reported result (*https://nested-knowledge.com/nest/rob/912*). Upon conducting a visual inspection of the funnel plots (refer to Figs S9–S12), we noticed asymmetry for certain outcomes such as LOS, ICA, costs, and radiation exposure. Also, the Egger test was statistically significant for ICA (*P* = .04), revascularization (*P* = .005), and LOS (*P* = .04). While this could suggest the possibility of publication bias, it is important to note that it may also be a result of true heterogeneity among the included studies (37). Also, the certainty of the evidence was rated as high by the GRADE system for all outcomes (Table S2).

## Discussion

This LSR and meta-analysis reassures health care decision makers that CCTA is a safe strategy to rule out ACS in adult patients presenting with ACP as pooled evidence shows similar incidence of MI (RR, 0.86; 95% CI: 0.66, 1.12) and mortality (RR, 0.96; 95% CI: 0.59, 1.58) between CCTA and SOC arms. Moreover, the use of CCTA is associated with reduced LOS (17%; 95% CI: 8%, 26%) and short-term costs (21%; 95% CI: 10%, 30%) in low- to intermediate-risk cohorts but not in high-risk patients which supports the recommendation of current chest pain guidelines (4,38). However, it is worth noting that this LSR did not evaluate the cost of downstream investigations for incidental findings due to the absence of comprehensive trial data.

The ROMICAT-II (19) and ACRIN-PA (18) studies were the major contributors to the observed reduction in LOS in participants presenting with ACP. These studies enrolled participants in the ED with scanners and CCTA reports readily available which may have contributed to reduced LOS. However, the studies were performed before the era of high-sensitive troponins, and studies incorporating this new tool showed shorter LOS in SOC arms and no difference compared with CCTA arms (11,28). Also, this reduction in LOS seems to be more important in the subgroup of participants with normal coronaries or nonobstructive CAD, since they can be securely discharged at a faster pace compared with those undergoing SOC. On the other hand, participants with obstructive CAD did not experience a different LOS compared with SOC arms given the necessity of additional testing to confirm ACS. Thus, it is expected that studies with individuals bearing higher pretest probability for ACS will diminish the effects of CCTA in decreasing LOS. This is supported by our findings which revealed no evidence of a difference in LOS between CCTA and SOC arms in studies containing greater than or equal to 10% of high-risk participants. Of note, one of the studies in this group (12) randomized participants during their original visit at the ED, hospital, or cardiology unit but allowed CCTA to be performed either during that visit or after discharge within 72 hours of randomization. This study revealed a 10% increase in the LOS for the CCTA arm (95% CI: 0%, 21%). These contrasting results underscore the importance of appropriate patient selection and the necessity to increase availability and timeliness of CCTA.

Our analysis confirms that using a CCTA-based strategy for triaging patients with ACS can reduce short-term costs. This is likely due to several factors including a decrease in LOS for participants with low-to-intermediate risk as well as fewer hospitalizations and less additional stress testing compared with the SOC group when the attending physicians have discretion in ordering further tests. It is noteworthy that the CCTA arm exhibited a slight rise in the number of revascularizations and medication adjustments, especially among participants in the low-to-intermediate risk group and when the SOC mandated stress echocardiography or nuclear medicine studies. A plausible explanation of this finding could be the capabilities of CCTA to provide enhanced anatomic visualization of the coronary tree, resulting in better selection of patients requiring revascularization or initiation of preventive medical therapy. Indeed, a subanalysis study of ACRIN-PA (32) demonstrated that in general, participants without stenosis undergoing CCTA versus SOC were less likely to be prescribed medications, whereas those with stenosis had a higher likelihood of starting medications. In the scenario of stable chest pain, the use of CCTA has been associated with increased use of both preventive therapies and coronary revascularization, probably due to the better characterization of CAD (39). Also, these changes in medication were associated with reduced rates of subsequent death from coronary heart disease or nonfatal MI (40). CCTA may also overestimate the degree of stenosis, especially in patients with heavy coronary calcification (41), which in turn could result in unnecessary downstream procedures. The available information can neither confirm nor refute these hypotheses, nor does it provide insight on whether additional revascularizations were associated with better clinical outcomes.

The results of this LSR suggest that hard clinical outcomes such as MI and mortality are not affected by the choice of ACP evaluation strategy. Newer CT techniques such as CT stress perfusion or CT fractional flow reserve, which can be performed concurrently with CCTA, may improve the specificity and positive predictive value, allowing for better identification of lesions with functional significance (42). This strategy could also further contribute to the reduction of ICA examinations and unnecessary revascularizations by identifying the hemodynamic significance of incidental coronary stenosis, further decreasing overall resource utilization and health care costs.

Our data about incidental findings with CCTA are limited to one study (35) which showed increased incidental findings contributing to increased in-hospital workup compared with SOC. Such increases could ultimately lead to longer LOSs (43). However, most incidental findings are non–life-threatening or unimportant and few cases require additional follow-up, being manageable during the regular outpatient workup (44).

One of the major concerns with CCTA is the radiation exposure it involves. In our study, we found that participants at high risk for ACS were exposed to increased radiation, possibly due to the higher prevalence of CAD. This often leads to additional tests using nuclear medicine stress perfusion, which further exposes patients to radiation. Moreover, although we did not have enough data to run a meta-regression for this outcome, it is worth noting that the type of stress test used in SOC plays a crucial role in radiation exposure, as exercise bicycle and treadmill tests or stress echocardiography do not expose patients to radiation, while nuclear medicine tests do. Fortunately, emerging technologies are making substantial contributions to reducing the radiation dose at CCTA examinations. For instance, artificial intelligence iterative reconstruction has the potential to further reduce radiation exposure, while CT fractional flow reserve could increase its specificity, thereby avoiding the need for additional stress testing (42).

Our study had limitations. Although we pooled estimates for LOS and costs, it is important to note that there was a high level of heterogeneity in the metrics used for these measures across the studies. This variability limits the generalizability of the pooled estimates. Consequently, we urge caution in interpreting these results and recommend considering the specific context and metrics of each study when evaluating LOS and costs. Additional studies investigating the effects of coronary artery calcium score or CT fractional flow reserve for triaging patients with ACP were not included, as this would require a different search query to identify all related studies; therefore, this should be investigated with a separate meta-analysis. However, these measures might affect multiple outcome parameters, including but not limited to the LOS, costs, rate of further testing, and rate of revascularization.

In conclusion, our results support the current guidelines’ recommendations for the use of CCTA as a safe, rapid, and less expensive in the short term strategy to exclude ACS in low- to intermediate-risk patients presenting with ACP.
